# Iron supplementation inhibits hypoxia-induced mitochondrial damage and protects zebrafish liver cells from death

**DOI:** 10.3389/fphys.2022.925752

**Published:** 2022-08-26

**Authors:** Ruiqin Hu, Genfang Li, Qianghua Xu, Liangbiao Chen

**Affiliations:** ^1^ International Joint Research Centre for Marine Biosciences (Ministry of Science and Technology), College of Fisheries and Life Science, Shanghai Ocean University, Shanghai, China; ^2^ Key Laboratory of Exploration and Utilization of Aquatic Genetic Resources (Ministry of Education) and International Research Centre for Marine Biosciences, College of Fisheries and Life Science, Shanghai Ocean University, Shanghai, China; ^3^ Key Laboratory of Sustainable Exploitation of Oceanic Fisheries Resources, College of Marine Science, Shanghai Ocean University, Shanghai, China

**Keywords:** hypoxia, iron, ROS, mitochondria, mitophagy

## Abstract

Acute hypoxia in water has always been a thorny problem in aquaculture. Oxygen and iron play important roles and are interdependent in fish. Iron is essential for oxygen transport and its concentration tightly controlled to maintain the cellular redox homeostasis. However, it is still unclear the role and mechanism of iron in hypoxic stress of fish. In this study, we investigated the role of iron in hypoxic responses of two zebrafish-derived cell lines. We found hypoxia exposed zebrafish liver cells (ZFL) demonstrated reduced expression of Ferritin and the gene *fth31* for mitochondrial iron storage, corresponding to reduction of both intracellular and mitochondrial free iron and significant decrease of ROS levels in multiple cellular components, including mitochondrial ROS and lipid peroxidation level. In parallel, the mitochondrial integrity was severely damaged. Addition of exogenous iron restored the iron and ROS levels in cellular and mitochondria, reduced mitochondrial damage through enhancing mitophagy leading to higher cell viability, while treated the cells with iron chelator (DFO) or ferroptosis inhibitor (Fer-1) showed no improvements of the cellular conditions. In contrast, in hypoxia insensitive zebrafish embryonic fibroblasts cells (ZF4), the expression of genes related to iron metabolism showed opposite trends of change and higher mitochondrial ROS level compared with the ZFL cells. These results suggest that iron homeostasis is important for zebrafish cells to maintain mitochondrial integrity in hypoxic stress, which is cell type dependent. Our study enriched the hypoxia regulation mechanism of fish, which helped to reduce the hypoxia loss in fish farming.

## Introduction

Dissolved oxygen is one of the key physical and chemical factors in aquatic ecological environment, and also is an important limiting factor in fish culture (Valavanidis et al., 2006). Aquatic habitats often experience extreme fluctuations in O_2_ content ranging from near anoxia (<1% O_2_) to hyperoxia (300–500% O_2_) reflecting the local dynamics of the photosynthesis, respiration and atmospheric gas exchange (Diaz and Rosenberg, 2008). Although terrestrial amphibians and reptiles encounter hypoxic burrow environments, the degree of hypoxia that experienced by aquatic species is more serious (Bickler and Buck, 2007). Low dissolved oxygen, or hypoxia, can negatively affect fish behavior, physiology, immunology, and growth (Abdel-Tawwab et al., 2019; Qiang et al., 2019). Mild hypoxic conditions do not cause fish death, although it can cause behavior and feeding abnormalities, but severe hypoxic conditions or anoxia (0.1% O_2_) can be fatal to fish (Richards, 2011). Studies found that red blood cells (RBC), hemoglobin and/or blood cells in fish increased rapidly under hypoxia stress (Affonso et al., 2002; Boggs et al., 2022). Hemoglobin is an important protein that carries oxygen in red blood cells. Thus, more hemoglobin needs to be synthesized to increase oxygen supply under hypoxic conditions.

Iron is essential for oxygen transport and is a component of proteins that carry oxygen molecules, such as hemoglobin and myoglobin, which iron deficiency causes anemia, limits mitochondrial respiration and result in mitochondrial DNA damage (Walter et al., 2002; Rouault 2006; Salahudeen and Bruick, 2009). At the physiological level, cellular iron is necessary for maintenance of metabolism, and excessive free iron may lead to oxidative damage and/or cell death (Mittler 2017). Therefore, the homeostasis of iron must be strictly controlled. Many studies have shown that hypoxia stress causes iron metabolism dysfunction, and hypoxia can also protect cells from damage caused by the disrupted iron metabolism (Fuhrmann et al., 2020; Duarte et al., 2021; Ni et al., 2021). It is very important to coordinate the physiological hypoxia response with the effective control of iron. However, it is still unclear the role and mechanism of iron in hypoxic stress of fish.

Superoxide and other reactive oxygen species (ROS) have been recognized harmful and toxic byproducts of aerobic metabolism, but they are also important signaling molecules in a variety of physiological and pathophysiological conditions (Sena and Chandel, 2012; Mittler 2017). Low level of ROS is utilized for signal transduction, but prolonged elevations of ROS result in the oxidation of nucleic acid, protein, lipid and leading to cell dysfunction or death (Zhang et al., 2008). ROS and ROS-dependent signaling pathways seem to be linked in different ways to those that adapt to low oxygen conditions (Schieber and Chandel, 2014). As one of the significant source of cellular ROS, mitochondria play central roles in hypoxic responses (Lenaz 2001; Murphy 2009; Zhang and Wong, 2021). On one hand, mitochondria themselves are especially vulnerable to ROS-mediated oxidative damage (Sena and Chandel, 2012). On the other hand, ROS production from the mitochondria is temporarily increased in response to acute hypoxia (Hernansanz-Agustín et al., 2014). However, localization and speciation of the paradoxical increase in reactive oxygen species production in hypoxia remain debatable. Therefore, the cell types and the time frame of hypoxic should be considered.

Mitochondria have been viewed as pluripotent organelles, controlling cellular life, stress and death (Galluzzi et al., 2012). Thus, maintaining a functional mitochondrial network is the basis of cellular homeostasis in response to conditioned stress and physiological adaptation. Mitochondrial autophagy is one of the major mechanisms of mitochondrial quality control (Ashrafi and Schwarz, 2013; Ni et al., 2015). Autophagosomes selectively engulf dysfunctional or redundant mitochondria and degrade them in lysosomes (Kroemer and Jaattela, 2005). Mitophagy is highly sensitive to dynamic changes in endogenous metabolites, including iron-, glycolysis-TCA-, NAD + -, amino acids-, and fatty acid-related metabolites (Ting Zhang et al., 2021). In addition, disturbances of iron homeostasis (including iron deposition and iron deficiency) and abnormal iron metabolism have been widely reported to be closely associated with mitochondrial dysfunction (Bogdan et al., 2016; Wei et al., 2020). Mitochondria provide a place for iron metabolism in cells, and iron deprivation triggers mitophagy (Palikaras et al., 2018).

Fish liver is one of the earliest and most sensitive tissues to external stimuli, and is also the earliest tissue to appear damage (Kietzmann 2019). Meanwhile, liver plays an important role in iron homeostasis (Rishi and Subramaniam, 2017). Oxygen is the basic element to maintain cell life activities, and hypoxia will cause different effects on different cells (Michiels 2004). Therefore, we used zebrafish liver cells (ZFL) and zebrafish embryonic fibroblasts cells (ZF4), a kind of cell that is insensitive to both hypoxia and iron, to explore the role and mechanism of iron in fish cells respond to hypoxia stress. Hypoxia (3–0.1% O_2_) is capable of rapidly inducing, via the hypoxia-inducible factor (HIF-1), a cell survival response engaging autophagy. This process is a HIF-1dependent autophagic response which also mediate mitophagy, a metabolic adaptation for survival that is able to control reactive oxygen species (ROS) production. In contrast, severe hypoxic condition or anoxia (0.1% O_2_), where the latter is often confused with physiological hypoxia, are capable of inducing a HIF independent autophagic response. (Mazure and Pouysségur, 2010). Many studies focus on oxygen concentration above 1%, but there are few studies on lower oxygen concentration. Therefore, 0.1% oxygen concentration was selected as hypoxic stress for study. In this study, we demonstrated that hypoxic stress caused iron loss in the cytoplasm and mitochondria of ZFL cells, resulting in mitochondrial damage and ultimately cell death. The cell death due to hypoxia is a non-ferroptosis form of death, although iron supplementation can reverse the course. However, iron has different roles in ZF4 cells to respond to hypoxia stress. This study established the role of iron in maintaining mitochondrial integrity in hypoxic response, which help to understand the regulatory mechanism of fish response to hypoxia stress to reduce the hypoxia loss in fish farming.

## Material and method

### Cell culture and treatment

ZFL and ZF4 cells were provided by the Cell Bank of the Chinese Academy of Sciences. Cells were cultured in DMEM F12 medium containing 10% fetal bovine serum (FBS) and 100 U/mL penicillin/streptomycin in cell culture incubator (Eppendorf, Germany). The cells were seeded 24 h before to ensure attachment, then were cultured under normoxic (21% O_2_) and hypoxic (0.1% O_2_, balanced nitrogen) environment with 5% CO_2_ at 28°C when the cellular confluency reached 70%.

Biological reagents ferric ammonium citrate (FAC) (Sigma, United States), Deferoxamine (DFO) (MedChemExpress, United States) and Ferrostatin-1 (Fer-1) (MedChemExpress, United States) have been used to rescue cells under hypoxia stress. FAC, DFO and Fer-1 were dissolved and stored according to the manufacturers’ protocol, and then diluted to the appropriate concentration in cell culture medium for cell treatment. In detail, the final concentrations of FAC were 0, 0.1, 0.25, 0.5, 0.75, 1.0 and 2.0mM; the final concentrations of DFO were 0, 10, 30, 50, 70, 90 and 100µM; the final concentrations of Fer-1 were 0, 1, 2, 4, 6, 8 and 10 µM. Cells were pretreated with FAC, DFO and Fer-1 for 1 h before hypoxia stress.

### The determination of cell morphology

Cells were seeded onto 6 cm culture dishes with the cell confluency reached 70%. The cells were treated with or without FAC, DFO or Fer-1 for 1 h prior hypoxic incubations. Upon incubated with or without treatment for corresponding time under normoxia or hypoxia, the morphological features of cells were captured using Zeiss microscope directly.

### Cell vitality assay

To analyze the viability of the cells, cells were plated in 96-well plate with 90 µl medium 24 h before to ensure attachment and were cultured under normoxic (21% O_2_) environment with 5% CO_2_ at 28°C. When the cellular confluency reached 70%, cells in the control group were directly analyzed for cell activity, and cells in the experimental group were pretreated with FAC, DFO and Fer-1 for 1 h under normoxic (21% O_2_). Then the experimental group cells were transferred to hypoxic (0.1% O_2_, balanced nitrogen) environment with 5% CO_2_ at 28 °C for 1, 2, 3 and 4 d.

PrestoBlue™ HS Cell Viability Regent (Invitrogen, United States) were used to analyze the viability of the cells. Briefly, add 10 µl of cell viability regent directly to cells in culture medium, and incubate for 3 h under normoxic (21% O_2_) environment with 5% CO_2_ at 28 °C. Afterwards, absorbance was measured on a plate reader (Biotek, United States) at 570 nm, using 600 nm as a reference wavelength.

### Western blotting

Total protein was extracted using RIPA (sigma, Germany) lysis buffer containing protease inhibitor PMSF (Invitrogen, United States). Protein concentration was detected by BCA protein assay kit (Invitrogen, United States). Total of 20 µg protein was electrophoresed in 10% SDS-PAGE gels and transferred onto PVDF membranes (Merk, Germany). The membranes were blocked with TBST solution containing 5% milk for 2.5 h at room temperature, then probed with the primary antibody overnight and the secondary antibody for 1 h. Wash the membranes with TBST solution three times for 5 min each before and after incubation of secondary antibodies. Finally, the blots were captured by Amersham imager 600 (GE, United States) and the quantitative results were analyzed by ImageJ analysis software. The list of antibodies is as follows: Ferritin (Huabio, China), Hif1α (Boster, China), LC3 (Cell Signaling Technology, United States), P62 (Cell Signaling Technology, United States), Actin (Huabio, China) and the secondary antibody (Huabio, China).

### Real-time quantitative PCR analysis

RNA was isolated using Trizol according to the manufacturer’s protocol (Invitrogen, United States) and measured using a Nanodrop spectrophotometer (ThermoFisher, United States). Reverse transcription was performed with the Maxima First Strand cDNA Synthesis Kit for RT-PCR (Takara, Japan). RNA expression of *tfa*, *tfr2*, *fth27*, *fth28* and *fth31* was analyzed using SYBR Green Master Mix (Roche, Switzerland) on a Roche 480 System (Roche, Switzerland) and normalized to Actin. Primers are listed in Table 1.

**TABLE 1:** Primer information.

Gene name	Forward primer	Reverse primer
*tfa*	AGC​AGC​AGA​CAT​TGA​GTG​TC	TTT​GCT​CCA​TCT​ACT​GTA​AC
*tfr2*	AGC​AGT​TTA​CCT​CAC​ACT​GAC	AGG​AAT​GTT​GTC​CGG​CTC​G
*fthl27*	TGC​GAG​GCT​TTG​ATC​AAC​AAG	TGG​CAA​ATC​CAG​GAA​GAG​CC
*fthl28*	AAG​ATG​ATC​AAT​CTG​GAG​C	TTG​AAG​AAC​TTG​GCA​AAT​CC
*fthl31*	AGG​CTG​CGA​TCA​ACA​AGA​TG	AGGAAGAGCCACATCGTC
*actin*	TGT​CCC​TGT​ATG​CCT​CTG​GT	AAGTCCAGACGGAGGATG

### Intracellular ROS measurement

Intracellular ROS production was detected by 2,7-dichlorodihydrofluorescein diacetate (CM-H2DCFDA) (Invitrogen, United States) according to the manufacturer’s introduction. Briefly, ZFL and ZF4 cells were seed in 6 cm plates and harvested after treatment for 3 days. Firstly, cells were washed twice with DPBS solution (Sangon, China). Then cells were labeled with 5 μM CM-H2DCFDA for 30 min at 28°C and washed for two times. The fluorescence was quantified using Biosciences AccuriC6 flow cytometry (Becton Dickinson, United States) with an excitation wavelength of 488 nm and emission at 525 nm.

### Detection of lipid peroxidation level

Lipid peroxidation was quantified by incubating with C11-BODIPY™ superoxide indicator (Invitrogen, United States). This fluorophore is readily incorporated into cellular membranes and is about twice as sensitive to oxidation as arachidonic acid, thereby losing its bright red fluorescence with a shift to green. The green fluorescence was selectively detected using the excitation and emission bandpass filters of 488 and 510 nm, respectively. Therefore, the increase of oxidative stress was accompanied with a linear increase in green fluorescence intensity.

In brief, cells were washed twice with DPBS solution firstly, then incubated with C11-BODIPY reagent solution (5 μM) at 28°C for 15 min. Removed the remaining C11-BODIPY reagent and washed cells two times with DPBS solution. The fluorescence was quantified using Biosciences AccuriC6 flow cytometry (Becton Dickinson, United States) at the fluorescence intensity (488/510 nm).

### Mitochondria-derived ROS determination

The level of mitochondria-derived ROS is determined with the MitoSOX™ Red mitochondrial superoxide indicator (Invitrogen, United States). Briefly, cells were washed twice with DPBS solution firstly, then incubated with MitoSOX reagent solution (5 μM) at 28°C for 15 min. Removed the remaining MitoSOX reagent and washed cells two times with DPBS solution. The fluorescence was quantified using Biosciences AccuriC6 flow cytometry (Becton Dickinson, United States) at the fluorescence intensity (510/580 nm).

### Mitochondrial iron determination

Mitochondrial iron concentration was determined by RPA (rhodamine B-[(1,10-phenanthrolin-5-yl)aminocarbonyl] benzyl ester) (Squarix biotechnology, Germany). Deprotonated rhodamine B (rhodamine B base) was chosen for mitochondrial targeting and as a fluorophore, and was coupled with 4-(bromomethyl)-N-(1,10- phenanthrolin-5-yl) benzamide for iron chelation, which is the specific indicator allowing selective determination of mitochondrial chelatable iron in viable cells (Bapat et al., 2002; Gordan et al., 2020).

In brief, ZFL cells were washed with DPBS solution for two times and incubated with RPA dye solution (10 μM) at 28°C for 15 min. Cells were removed remaining RPA reagent and washed for two times with DPBS solution. Then photographed with Zeiss fluorescence confocal microscope (Zeiss, Germany) at proper fluorescence intensity (564/601 nm) and the density of fluorescence was analyzed by ImageJ analysis software (Tang et al., 2019).

### Intracellular iron determination

Intracellular iron concentration was determined by PGSK fluorescence probe (Phen Green™ SK, Diacetate) (Invitrogen, United States). PGSK fluorescence is quenched upon binding chelatable iron and fluorescence intensity can be used to quantify the amount of chelatable iron when the remaining probe is removed (Petrat et al., 1999; Ramachandran et al., 2004; Siri-Angkul et al., 2021).

Briefly, ZFL cells were washed with DPBS solution for two times and incubated with PGSK dye solution (10 μM) at 28°C for 15 min. Cells were removed remaining PGSK reagent and washed for two times with DPBS solution. Ferrous ion in cytoplasm could quench the green fluorescence of cell. Then photographed with Zeiss fluorescence confocal microscope at proper fluorescence intensity (507/532 nm) and the density of fluorescence was analyzed by ImageJ analysis software.

### RNA sequencing and analysis

Total RNA of ZFL cell was extracted using TRIzol Reagent according to the manufacturer’s protocol (Invitrogen, United States). The concentration of total RNA was determined with a Qubit fluorometer (Life Technologies, United States). A microgram of RNA from each sample was used to prepare the mRNA-Seq library with the TruSeq RNA Sample Prep Kit (Illumina, United States) following the manufacturer’s instructions and then sequenced with Illumina HiSeq 2,500 system.

RNA-seq reads were trimmed using Trimmomatic (Ver. 0.33 AVGQUAL:20 TRAILING:20 MINLEN:50). The clean Illumina paired-end reads of each sample were mapped to the annotated zebrafish genome (GRCz11) using HISAT2 aligner (Ver. 2.0.4). Cufflinks was used to count the reads for each gene and transformed to FPKM. Differentially expressed genes (DEGs) were determined using the edgeR package developed in R. Genes with log2FC > 1 and *p*_value <0.05 were up-regulated. Genes with log2FC < -1 and *p*_value <0.05 were down-regulated, GO enrichment and KEGG enrichment analysis for the DEGs were performed using ClusterProfiler package (cutoff, *p*-value < 0.05). Volcanic maps were generated using R.

### Analysis of mitochondrial membrane potential

Mitochondrial membrane potential of ZFL cells were measured by JC-1 fluorescent probe (Beyotime, China). After treatment, cells were incubated with JC-1 working solution at 28°C for 30 min. Subsequently, cells were washed with JC-1 buffer solution for two times. Then photographed with Zeiss fluorescence confocal microscope at proper fluorescence intensity (485 and 590 nm). Results were expressed as the ratio of the red/green fluorescence intensity, which represented the degree of mitochondrial damage.

### Mitochondrial tracking

Cells were plated on coverslips. After treatment, remove the medium from the dish and add the prewarmed MitoTracker probe-containing medium (Invitrogen, United States). Incubate the cells for 30 min at 28°C. Then replace the loading solution with fresh 4% PFA (Sangon, China) for 15 min. Washed cells two times with DPBS solution and photographed with Zeiss fluorescence confocal microscope at proper fluorescence intensity (644/665 nm).

### Lysosomal tracking

Cells were plated on coverslips. After treatment, remove the medium from the dish and add the prewarmed LysoTracker™ Red DND-99 probe-containing medium (Invitrogen, United States). Incubate the cells for 1 h at 28°C. Then replace the loading solution with fresh 4% PFA for 15 min. Washed cells two times with DPBS solution and photographed with Zeiss fluorescence confocal microscope at proper fluorescence intensity (577/590 nm).

### Statistical analysis

All data was performed at least three times. Bar graphs were plotted, and error bars were calculated using GraphPad Prism seven software (San Diego, United States). Statistical analysis was conducted using the Student’s t-test or one-way ANOVA with Tukey’s post hoc test. All values are shown as mean ± s.d., *p* < 0.05 were considered statistically significant. One asterisk, two asterisks and three asterisks indicate *p* < 0.05, *p* < 0.01 and *p* < 0.001, respectively.

## Results

### Hypoxia inhibits cell growth and ROS production, leading to iron deficiency in cytoplasm and mitochondria

**FIGURE 1:**
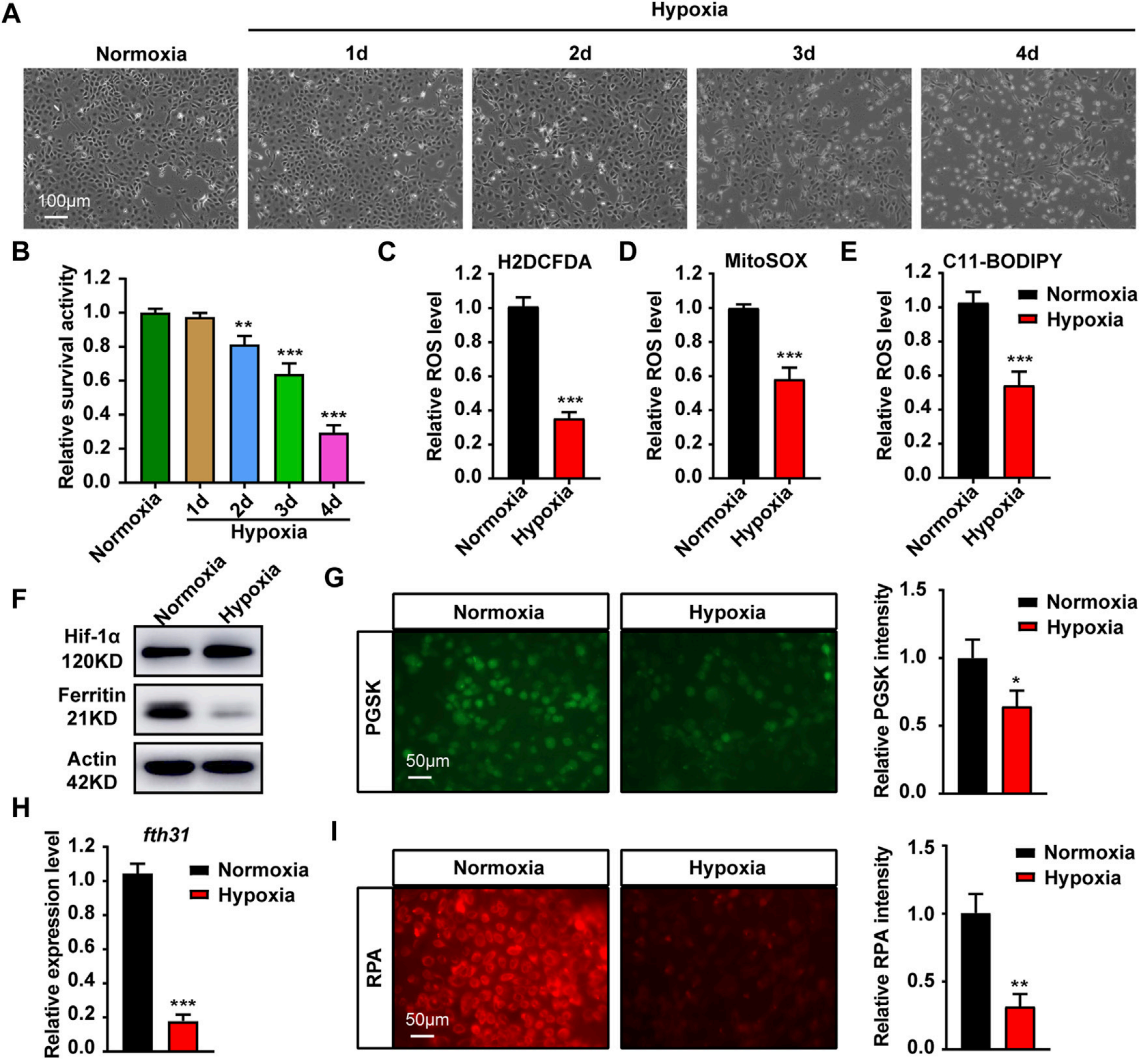
Hypoxia stress affects cell growth, ROS production and iron metabolism in cytoplasma and mitochondria. **(A)** Microscopic analysis of morphological changes of ZFL cells under normoxia or hypoxia for 1, 2, 3 and 4 d. **(B)** Cell Viability analyzed with PrestoBlue™ HS Cell Viability Regent under normoxia or hypoxia for 1, 2, 3 and 4 d. **(C–E)** Analysis of changes in total ROS **(C)**, mitochondrial-derived ROS **(D)** and lipid peroxidation **(E)** levels in cells with CM-H2DCFDA, MitoSOX and C11-BODIPY probe under normoxia and hypoxia for 3 days. **(F)** Western blot analysis of Ferritin expression in ZFL cells cultured under normoxia and hypoxia for 3 days. **(G)** Phen Green™ SK (PGSK) probe analysis and quantification of cytoplasmic free iron content in ZFL cells after treated under normoxia and hypoxia for 3 days. **(H)** The mRNA expression of mitochondrial iron storage gene *fth31* in ZFL cells quantified by real-time RT–PCR under normoxia and hypoxia for 3 days. **(I)** Fluorescence microscope with RPA red indicator analysis and quantification of mitochondrial iron content in ZFL cells treated under normoxia and hypoxia for 3 days. Normoxia was used as a control group for significance analysis. Error bars, mean ± s.d., *n* = 3 (biological replicates).

We first investigated the effects of hypoxia on ZFL cells. At 21% O_2_ (normoxia), ZFL cells grew well. When grew at 0.1% O_2_ (hypoxia), however, cells grew poorly and began to die severely from the third day, with only a small fraction of the cells (about 20%) survived to the fourth day (Figures 1A,B). We then investigated the effect of hypoxia on ROS levels, since the main effect of hypoxia is oxidative damage. We examined the total ROS level by CM-H2DCFDA probe and found that ROS level was down-regulated under hypoxia stress (Figure 1C). Further investigation revealed that both mitochondrial-derived ROS and lipid peroxidation levels were reduced (Figures 1D,E). These results revealed that hypoxia is harmful to ZFL cells that can inhibit cell growth and ROS production.

Iron is the main driving force of REDOX reaction (Jomova and Valko, 2011). Western blotting showed that the expression of Ferritin, a cytoplasmic iron storage protein, was significantly reduced, suggesting loss of iron in the cytoplasm (Figure 1F). The same observation is also confirmed by chelatable Fe^2+^ sensitive probe PGSK, showing a significant reduction in chelatable iron (Figure 1G). Mitochondrial iron is required to trigger the ROS-oxidative stress via Fenton action (Levi and Rovida, 2009). The results revealed the expression of mitochondrial iron storage gene *fth31* was significantly reduced (Figure 1H), which was consistent with the results of specific probing of mitochondrial Fe^2+^ using RPA (Figure 1I). Collectively, hypoxia in the ZFL cells caused cytoplasmic and mitochondrial iron loss.

### Iron homeostasis is important for ZFL cells to respond to hypoxic stress, which is independent of ferroptosis

**FIGURE 2:**
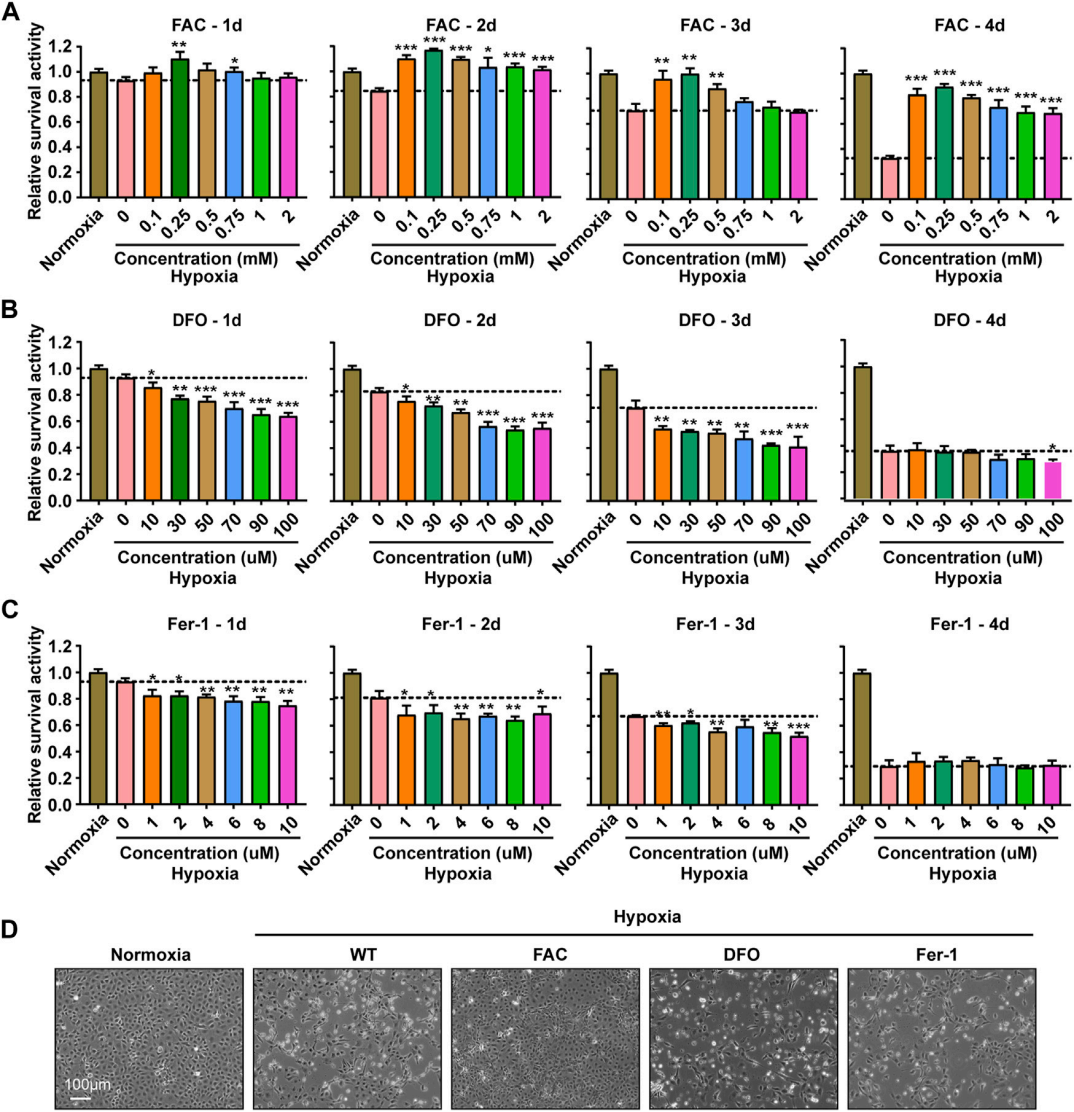
Iron supplementation could improve the survival activity of ZFL cells under hypoxia stress, but iron chelation and inhibition of ferroptosis could not. **(A–C)** Survival rate of ZFL cells analyzed with PrestoBlue™ HS Cell Viability Regent after treated with a series of concentrations of FAC **(A)**, DFO **(B)** and Fer-1 **(C)** under hypoxic stress from one to 4 days. Hypoxia 0 mM or hypoxia 0 µM was used as a control group for significance analysis. **(D)** The microscope analysis of morphology changes of ZFL cells under normoxia or treated with FAC (2.5 mM), DFO (10 µM) and Fer-1 (2.5 µM) under hypoxic stress for 3 days. Error bars, mean ± s.d., *n* = 3 (biological replicates).

To explore the effect of iron concentration on ZFL cell viability under hypoxia stress, FAC and DFO were used to supplement and chelate iron in ZFL cells respectively. The results showed that the proliferation capacity and survival rate of ZFL cells without FAC (0 mM) under hypoxia decreased gradually compared with the normoxia group (con group) (Figure 2A). However, the proliferation and survival rate of the hypoxic treated cells supplemented with proper amounts of FAC were significantly higher than that without exogenous iron supplementation (0 mM). Moreover, the proliferation and survival rate of FAC group (0.1, 0.25, 0.5 mM) were even higher than that of normoxia group after 2 days of hypoxia stress (Figure 2A). Evenly, the survival rate of ZFL cells in the FAC group was about 2–3 times higher on the fourth day of the treatment compared with the hypoxic group without FAC treatment (0 mM) (Figure 2A). Conversely, DFO treatment resulted in non-proliferation and significantly reduced cell survival compared with the normoxia group and the hypoxia control group (0 µM) in the first 3 days (Figure 2B), indicating iron chelation exacerbate ZFL death under hypoxic stress. At the fourth day, both DFO treated and untreated cells exhibited poor survival rates under hypoxia (Figure 2B).

Ferroptosis is an iron- and ROS-dependent form of regulated cell death (RCD) (Xie et al., 2016). To investigate whether the cell damage under hypoxia is related to ferroptosis, we tried to rescue the damage with Fer-1 (Ferrostatin-1), an inhibitor of ferroptosis by inhibiting lipid peroxidation. The results revealed that Fer-1 failed to rescue the hypoxia induced cell death, suggesting a ferroptosis independent death (Figure 2C). This observation was consistent with the decreased lipid peroxidation level (Figure 1E). Further morphological observation of cells showed that exogenous iron supplementation could prevent cell death and improve cell viability under hypoxia stress, while DFO and Fer-1 could not (Figure 2D).

### Iron supplementation restored ROS levels and reduced mitochondrial damage of ZFL cells under hypoxia

**FIGURE 3:**
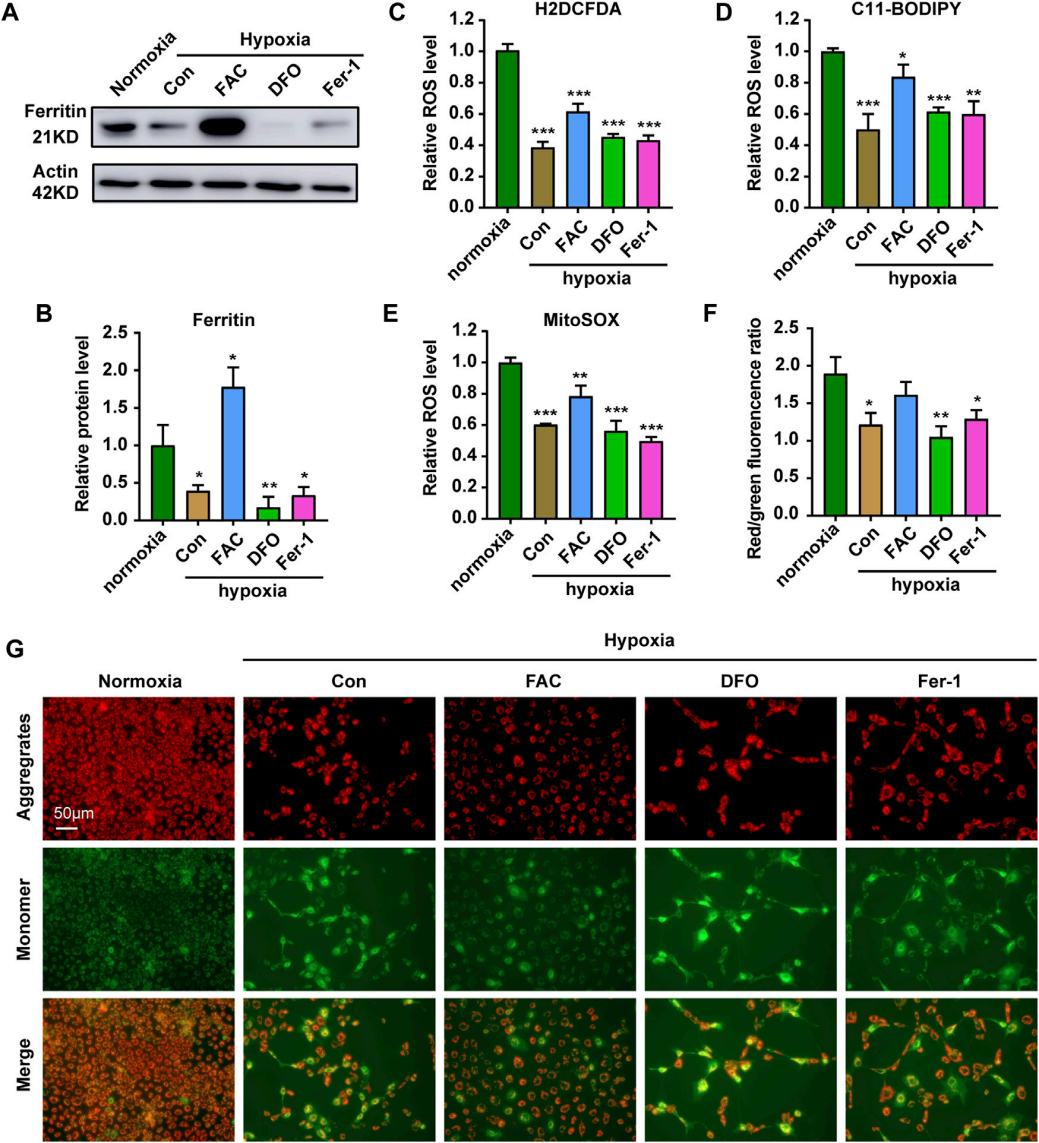
Iron supplementation can increase ROS level and reduce mitochondrial damage in cells under hypoxia stress. **(A,B)** Western blot analysis and quantification of Ferritin expression in ZFL cells cultured under normoxia and hypoxia treated with FAC, DFO and Fer-1 for 3 days. Actin as a loading control. **(C–E)** Analysis of changes in total ROS **(C)**, lipid peroxidation level **(D)** and mitochondrial-derived ROS **(E)** levels in cells with H2DCFDA, C11-BODIPY and MitoSOX probe under normoxia and hypoxia for 3 days. Cells under hypoxic stress were rescued with FAC (2.5 mM), DFO (10 µM) and Fer-1 (2.5 µM). **(F,G)** Quantitative results and representative images of cellular JC-1 fluorescence in normoxic cell and hypoxic cells treated with FAC (2.5 mM), DFO (10 µM) and Fer-1 (2.5 µM) under hypoxia stress. Normoxia was used as a control group for significance analysis. Error bars, mean ± s.d., *n* = 3 (biological replicates).

To explore the role of iron in the ZFL cells response to hypoxic stress, we carried out further exploration. Firstly, WB showed that exogenous iron supplementation increased the expression level of iron-storing protein Ferritin, while iron chelation reduced Ferritin level, whereas addition of the ferroptosis inhibitor had no significant effect (Figures 3A,B). Accordingly, further examination of ROS levels showed that only exogenous iron supplementation partially restored the total ROS levels (Figure 3C). As iron is the cofactor of lipoxygenase, iron supplementation significantly increased the level of lipid peroxidation (Figure 3D). In addition, mitochondria are important source of reactive oxygen species (ROS) (Lenaz 2001). Iron supplementations increase mitochondrial-derived ROS level (Figure 3E). Indeed, iron supplementation restored the membrane potential of mitochondria and reduced the damage, which did not occur in DFO nor Fer-1 treatment (Figures 3F,G). These results indicated the importance of iron in maintaining the mitochondrial integrity and a certain amount of ROS level to sustain the physiological function of mitochondria and cell under hypoxic stress.

### RNA-seq analysis revealed iron supplementation improved mitophagy

**FIGURE 4:**
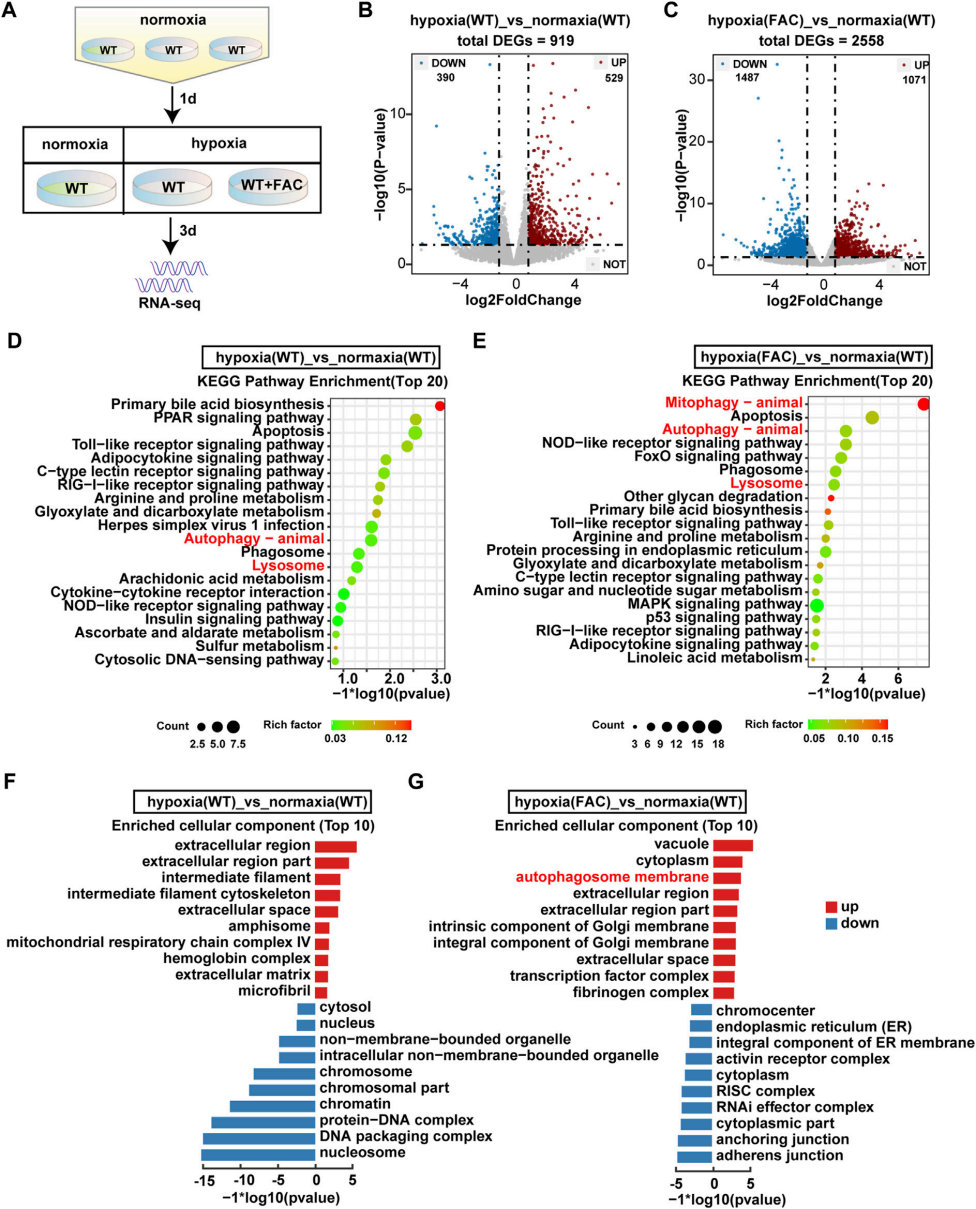
RNA-seq analysis revealed significant transecriptomic changes in hypoxia ZFL cell. **(A)** Flow chart of sampl processing for RNA-seq. WT: ZFL cell. WT + FAC: ZFL cell treated with FAC. **(B,C)** Volcanic map analysis of gene expression. UP: up-regulated differentially expressed gene; DOWN: down-regulated differentially expressed gene; NOT: non-differentially expressed gene. **(D,E)** The enriched KEGG pathways identified from up-regulated DEGs of hypoxia (WT) cell and hypoxia (FAC) cell compared with normoxia (WT) cell. **(F,G)** GO enrichment test on the DEGs of hypoxia (WT) cell and hypoxia (FAC) cell compared with normoxia (WT) cell.

In order to explore how the FAC protect ZFL cells from death under hypoxia stress, we performed RNA-seq analysis (Figure 4A). There were totally 919 and 2,558 genes that were differentially expressed in WT and FAC cells under hypoxia compared with WT cells under normoxia (Figures 4B,C). KEGG enrichment analysis revealed that the pathway of autophagy and lysosome were significantly up-regulated in WT hypoxia cells compared with WT normoxia cells (Figure 4D). However, the enrichment analysis of hypoxic cells treated by FAC found that mitophagy pathway was the most significantly up-regulated pathway except autophagy and Lysosome pathways (Figure 4E). Furthermore, GO enrichment analysis showed that autophagosome membrane components were significantly upregulated in FAC treated hypoxia cell (Figures 4F,G). Taken together, these analyses suggest that maintaining mitochondrial normality is essential for ZFL cells to survive the hypoxia stress.

### Iron supplementation restores mitochondrial function under hypoxia stress

**FIGURE 5:**
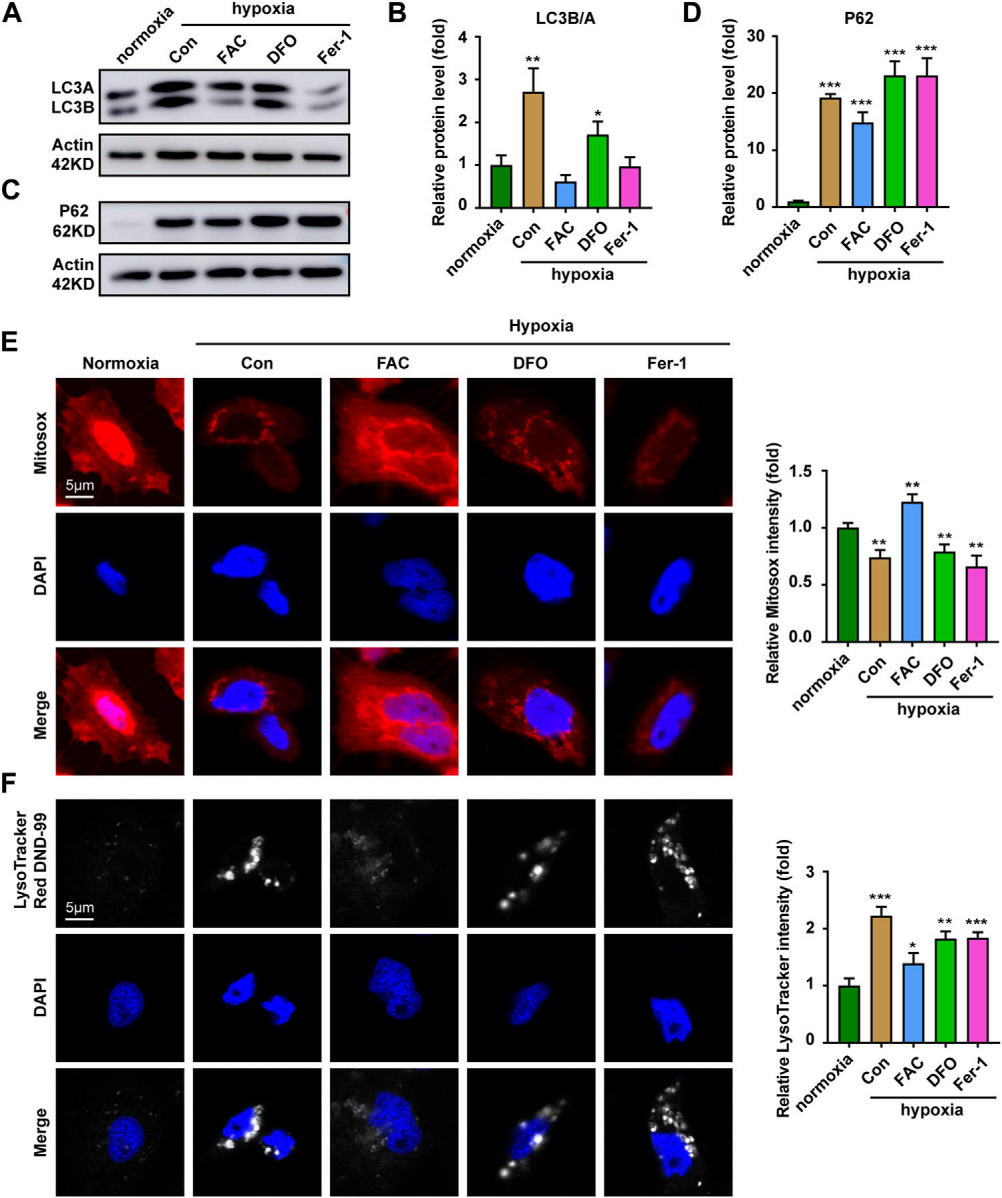
Iron supplementation increases the proportion of functioning mitochondria. **(A,B)** Western blot analysis and quantitative results of LC3 expression in ZFL cells cultured under normoxia and hypoxia treated with or without FAC (2.5 mM), DFO (10 µM) and Fer-1 (2.5 µM) for 3 days. Actin as a loading control. **(C,D)** Western blot analysis and quantitative results of P62 expression in ZFL cells cultured under normoxia and hypoxia treated with or without FAC (2.5 mM), DFO (10 µM) and Fer-1 (2.5 µM) for 3 days. Actin as a loading control. **(E)** Representative images and quantitative results of Mitotraker red Dye to visualize mitochondrial mass in normoxia cell and cell treated with or without FAC (2.5 mM), DFO (10 µM) and Fer-1 (2.5 µM) under hypoxia stress for 3 days. **(F)** Representative images and quantitative results of Lysotraker red DND-99 Dye to visualize acid lysosomal in normoxia cell and cell treated with or without FAC (2.5 mM), DFO (10 µM) and Fer-1 (2.5 µM) under hypoxia stress for 3 days. Normoxia was used as a control group for significance analysis. Error bars, mean ± s.d., *n* = 3 (biological replicates).

Mitochondrial quality is controlled by the selective removal of damaged mitochondria through mitophagy (Ashrafi and Schwarz, 2013). Results indicated the expression of autophagy-related proteins LC3 and P62 were significantly up-regulated under hypoxia stress (Figures 5A–D). In combination, the expressions of LC3 and P62 after iron supplementation were weaker than those of chelated iron and inhibited ferroptosis (Figures 5A–D). Further exploration found that mitochondria were evenly distributed in the cytoplasm under hypoxia supplemented with iron, while the density of mitochondria decreased and clustered around the nucleus treated with DFO and Fer-1 (Figure 5E). The tracking of lysosomes revealed that the level of acid lysosomes increased and showed a large amount of aggregation in the control group (Figure 5F), indicating that hypoxia caused mitochondrial damage (Figures 5E,F). However, iron supplementation could improve mitochondrial damage caused by hypoxic stress, while DFO and Fer-1 did not.

### ZF4 cells showed different patterns of iron responses under hypoxic condition

**FIGURE 6:**
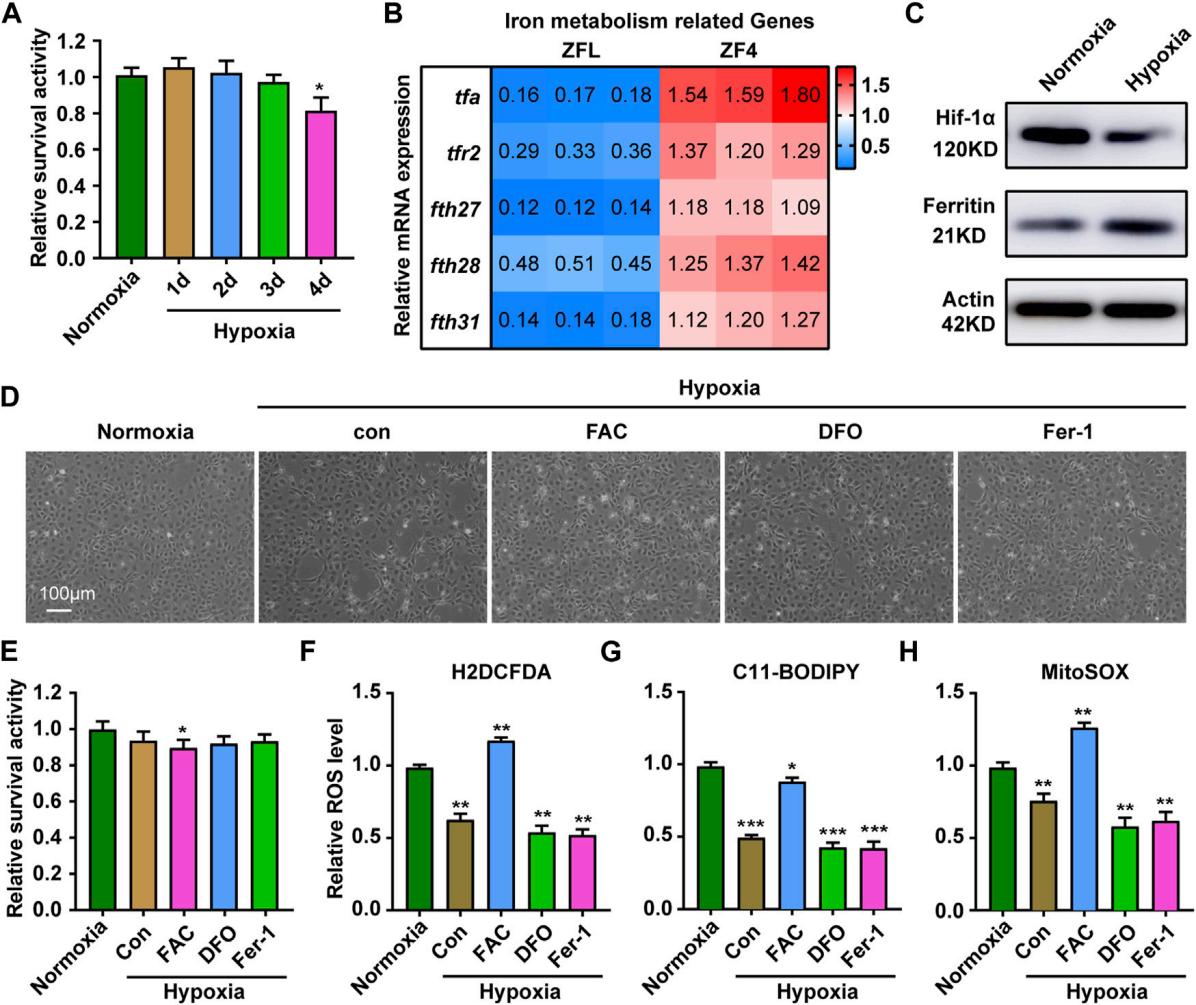
Response of ZF4 cells to hypoxic stress. **(A)** Cell Viability was analyzed with PrestoBlue™ HS Cell Viability Regent under normoxia or hypoxia treated for 1, 2, 3 and 4 d. **(B)** The mRNA expression of iron absorption and storage gene in ZFL cells was quantified by real-time RT–PCR under normoxia and hypoxia for 3 days. **(C)** Western blot analysis of Hif-1α and Ferritin expression in ZF4 cells cultured under normoxia and hypoxia for 3 days. **(D)** The microscope analysis of morphology changes in ZF4 cells under normoxia or hypoxia treated with or without FAC (2.5 mM), DFO (10 µM) and Fer-1 (2.5 µM) for 3 days. **(E)** Cell Viability was analyzed with PrestoBlue™ HS Cell Viability Regent under normoxia or hypoxia treated with or without FAC (2.5 mM), DFO (10 µM) and Fer-1 (2.5 µM). **(F–H)** Analysis of changes in general ROS **(F)**, mitochondrial-derived ROS **(G)** and lipid peroxidation **(H)** levels in cells with H2DCFDA, MitoSOX and C11-BODIPY probe under normoxia and hypoxia for 3 days. Cells under hypoxic stress were rescued with FAC (2.5 mM), DFO (10 µM) and Fer-1 (2.5 µM). Normoxia was used as a control group for significance analysis. Error bars, mean ± s.d., *n* = 3 (biological replicates).

Given that the liver is an important organ for iron metabolism (Rishi and Subramaniam, 2017), we next sought to test whether other cell types had similar responses. Results showed that compared with ZFL cells, ZF4 cells had a better survival rate under hypoxia stress, with about 80% cells being alive on the fourth day (Figure 6A). The mRNA expression of genes related to iron metabolism showed completely opposite trends in ZF4 cells (Figure 6B), and also the protein levels of Hif1a and Ferritin (Figure 6C), indicating cytoplasmic and mitochondrial iron contents was increased in this cell line. Consistent with this, iron supplementation reduced the cell survival rate under hypoxia, while iron chelation or inhibition of ferroptosis showed no significant changes (Figures 6D,E). Further ROS level detection of cells showed that hypoxia stress reduced the general ROS level (Figure 6F), mitochondrial-derived ROS and lipid peroxidation levels (Figures 6G,H). In addition, iron supplementation can significantly increase ROS level, and even mitochondrial ROS and general ROS level exceed those of normoxic group (Figures 6F,H), which caused cell damage (Figures 6D,E). Chelation of iron or inhibition of ferroptosis slightly lowers ROS level. These results suggest that ZF4 cells have a different hypoxic stress response in terms of iron regulation compared with ZFL cells, indicating iron regulation under hypoxic response is cell type dependent.

## Discussion

In this study, we revealed hypoxia causes iron loss in cytoplasm and mitochondria of the ZFL cells, which leads to mitochondrial damage and ultimately cell death. Iron supplementation inhibits hypoxia-induced cell death, increase ROS levels, reduce mitochondrial damage and restore mitochondrial function. Therefore, iron plays essential roles in mitochondrial responses to hypoxic stress in ZFL cells. The involvement of iron in hypoxia responses is also demonstrated by ZF4 cells in which higher cell survival rate is sustained with the elevation of expression of the iron storage proteins.

Oxygen and iron are important for the health of living organisms. Hypoxia imposes stress to cells and organisms, which occurs under both pathological and non-pathological conditions (Fuhrmann and Brune, 2017). Cells in living organisms adapt to hypoxia by changing metabolism, which is facilitated by changes in protein expression, mRNA or protein stability that occur at the level of transcription or translation (Solaini et al., 2010; Zhang et al., 2021). Multiple studies have clearly shown that oxygen homeostasis and iron metabolism are interlinked, such as the target of HIF, which play an important role in cell adaptation to low oxygen levels in normal and damaged tissues, are involved in iron homeostasis (Shah and Xie, 2014; Xu et al., 2017; Yang et al., 2018; Han et al., 2022). The regulation between hypoxia and iron is complexed. On the one hand, hypoxia stress can cause iron homeostasis disorder (Aral et al., 2020; Hu et al., 2022); on the other hand, hypoxia is beneficial and can protect cells from death caused by abnormal iron metabolism (Ast et al., 2019; Fuhrmann et al., 2020; Ni et al., 2021). Our study also confirms that maintaining iron homeostasis is critical to respond to hypoxia stress. The expression of genes related to iron absorption and storage was significantly decreased in ZFL cells under hypoxia stress, which directly correlated hypoxia with iron metabolism.

Mitochondria are the main consumers of oxygen in cells and are severely affected by reduced oxygen utilization (Hamanaka and Chandel, 2009; Dan Dunn et al., 2015). Mitochondria require large amounts of iron for heme synthesis and iron-sulfur cluster maturation to form the electron transfer chain essential for oxidative phosphorylation (Hirota 2019). Thus, iron metabolism is essential for mitochondrial function and cell survival. Controlling the balance between iron availability and physiological hypoxia response is important to maintain intracellular homeostasis (Lakhal-Littleton and Robbins, 2017). ROS produced by the OXPHOS complex not only manifests as an unexpected escape of electrons from ETC and transfer to molecular oxygen, but also recognized as an important medium of cellular physiological signaling to cope with hypoxia (Zhang et al., 2022). As we found, hypoxic stress reduced the free iron content of cytoplasmic and mitochondrial, thereby reducing the ROS levels of cytoplasmic and mitochondrial, which can be restored by iron supplementation. This suggests that the homeostasis of free iron and ROS levels is important to respond to hypoxia stress.

Mitochondrial ROS (mtROS) considered as an essential component of physiological cell communication (Shadel and Horvath, 2015). Large amounts of mtROS directly destroy proteins, lipids, and nucleic acids, while low levels of mtROS act as signaling molecules to adapt to stress (Sena and Chandel, 2012; Fuhrmann and Brune, 2017). Even lower levels of mtROS are required for normal cell homeostasis (Sena and Chandel, 2012). Therefore, mtROS are not categorically harmful (Kalogeris et al., 2014). In a hypoxic environment, mitochondrial biology is altered (Fuhrmann and Brune, 2017). Presumably, mitochondria try to reduce ROS formation to reduce the risk of macromolecular damage during hypoxia (Fuhrmann and Brune, 2017). Excessive mtROS promote mitochondrial dysfunction and inflammation leading to mitochondrial damage (Zhao et al., 2021). Mitophagy is an essential mitochondrial quality control mechanism that eliminates damaged mitochondria and the production of ROS (Su et al., 2022). Our study found that iron supplementation can reduce mitochondrial damage and restore ROS levels produced by mitochondria, thereby enhancing the ability of cells to respond to hypoxic stress. There is certainly a need to learn more about the communicating ability and distinct targets of ROS under hypoxia and explore how a gradual and time-dependent decrease of oxygen affects mitochondrial biology.

Several studies of different groups, including marine gastropods, fish, frog and turtles, have reported increases in antioxidants during hypoxia (Hermes-Lima and Zenteno-Savin, 2002). This response to increased antioxidant levels in low oxygen conditions is called preparation for oxidative stress (Oliveira et al., 2018). This adaptive mechanism refers to the increased expression and/or activity of antioxidant enzymes during hypoxia, which may attenuate the effects of increased ROS formation during hypoxia (Estrada-Cárdenas et al., 2021). However, it has been suggested that increased mitochondrial ROS formation induced by hypoxia may trigger the activation of antioxidant defenses during hypoxia limitation (Welker et al., 2013). Our study found that hypoxia stress for 3 days led to a decrease in mitochondrial ROS levels in ZFL cells. Therefore, further investigations should be directed to study the interplay between hypoxia, ROS production, antioxidant and tolerance to hypoxia.

In recent years, research on aquaculture shows that iron plays an important role in hypoxic response. The comparative transcriptomic analysis revealed abundant hypoxia response-related genes and their differential regulation mechanism in muscle and liver of different common carp strains under acute hypoxia, including HIFs (hypoxia-inducible factors), MAP kinase, iron ion binding, and heme binding (Suo et al., 2022). Morphological observation on three tilapia exhibited widespread hepatic and splenic inflammation with marked hemosiderin accumulations which is caused by direct tissue hypoxia and polycythemia-related iron deposition (Edwards et al., 2020). Gene ontology enrichment analyses revealed that genes up-regulated by hypoxia are primarily involved in cellular iron homeostasis in zebrafish larvae (Long et al., 2015). Therefore, targeting iron homeostasis is a potential strategy to protect aquaculture against environmental hypoxia. Our findings show that iron supplementation improves the ability of ZFL cells to resist hypoxic stress, which will facilitate future investigation on the hypoxia response mechanism and provide a solid theoretical basis for breeding projects in aquaculture.

## Data Availability

The raw data supporting the conclusions of this article will be made available by the authors, without undue reservation. All of the Illumina RNA sequencing data of this project have been deposited at NCBI under the accession no. BioProject PRJNA848069 (http://www.ncbi.nlm.nih.gov/sra/).
